# RNF168 is highly expressed in esophageal squamous cell carcinoma and contributes to the malignant behaviors in association with the Wnt/β-catenin signaling pathway

**DOI:** 10.18632/aging.202471

**Published:** 2021-01-25

**Authors:** Yunjiu Gou, Dacheng Jin, Shengliang He, Songchen Han, Qizhou Bai

**Affiliations:** 1 Department of Thoracic Surgery, Gansu Provincial Hospital, Lanzhou, People’s Republic of China

**Keywords:** RNF168, esophageal squamous cell carcinoma, ubiquitin, Wnt/β-catenin

## Abstract

E3 ubiquitin ligase RING finger protein 168 (RNF168) is one of the key proteins in DNA damage repair. Abnormal expression of RNF168 has recently been found in some tumors. However, the role of RNF168 in the development of esophageal squamous cell carcinoma (ESCC) has not been fully elucidated. Here we report that expression of RNF168 in esophageal squamous cell carcinoma is increased with respect to normal esophageal epithelial tissue. Notably, in ESCC patients, increased RNF168 expression was associated with tumor stage and depth of invasion. Knockdown of the RNF168 gene inhibited proliferation of esophageal cancer cells, promoted cell apoptosis, and interfered with cell movement, ultimately inhibiting tumor xenograft growth. Mechanistic studies showed that RNF168 influenced the malignant behavior of esophageal cancer cells by regulating the Wnt/ β-catenin signaling pathway. In addition, RNF168 expression was positively correlated with wingless-type MMTV integration site family member 3A (WNT3A) expression, and high expression of RNF168 and WNT3A predicted a low survival rate. In conclusion, our findings highlight the important role of RNF168 in ESCC tumorigenesis and provide new biomarkers and therapeutic targets for the treatment of ESCC.

## INTRODUCTION

Esophageal squamous cell carcinoma (ESCC) has high morbidity and mortality rates, and accounts for 90% of all esophageal cancers [1]. Surgical treatment has proven effective for early stage ESCC. However, when first diagnosed most patients have progressed to advanced stages or their tumor has already metastasized. This malignant behavior is closely related to the high proliferative and invasive ability of esophageal cancer cells [2]. Like other cancers, ESCC is associated with a variety of genetic and epigenetic changes [3] which correlate with heterogeneity of certain markers. Despite a deeper understanding of ESCC and the identification of more and more therapeutic targets, its cure rate has remained low, while mortality remains high [4, 5]. Therefore, it is necessary to understand in more detail the molecular mechanisms underlying the occurrence and progression of ESCC, to identify new biomarkers, and to develop new treatment strategies.

Ubiquitin (Ub), a highly conserved 76-amino acid protein which can bind covalently to substrate proteins to mark them for degradation and which regulates a variety of cellular events, has become the focus of recent investigations on targeted cancer treatment [6]. The DNA double strand break (DSB) is one type of DNA damage which, if not properly repaired, may disturb cellular physiology, leading to severe consequences such as tumorigenesis [7]. E3 ubiquitin ligase RING finger protein 168 (RNF168) is a key protein in DNA damage repair, and deficiency in RNF168-dependent DSB repair has been reported to play a role in radiosensitivity, immunodeficiency, and teratogenicity [8, 9]. Panier et al. [10] suggested that RNF168 binds to Ub via its UMI, MIU and LRM domains. Thorslund et al. [11] concluded that when a DSB occurs in DNA, RNF168 first binds via its N terminal end to the RNF8 ubiquitin protein, and is then recruited to the DSB. Kongsema et al. [12] reported that RNF168 can trigger the ubiquitination and degradation of FOXM1 and other proteins, increasing the sensitivity of tumor cells to chemotherapy drugs, suggesting that RNF168 acts as a potential oncogene. However, investigations on the role of RNF168 in ESCC are scarce. In this study, we demonstrate that RNF168 overexpression is characteristic of ESCC and contributes to its malignant behavior.

## RESULTS

### Expression of RNF168 in ESCC and normal esophageal epithelium

**Figure 1:**
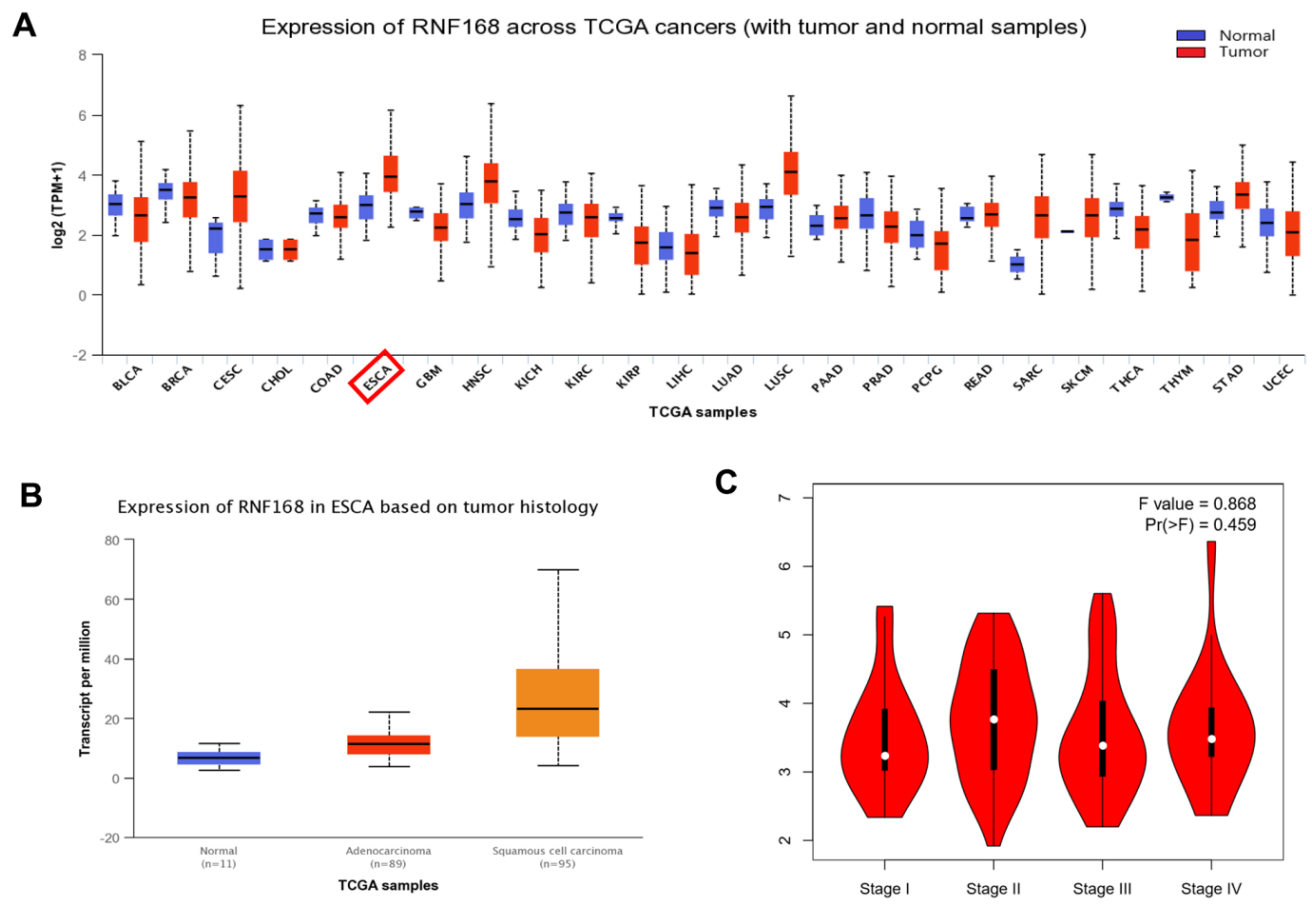
(**A**) Expression of RNF168 is altered in various types of tumors and is increased in ESCC; (**B**) RNF168 mRNA expression in ESCC tissues (N=182) and normal esophageal tissues (N=286) (*p<0.05); (**C**) Expression levels of RNF168 in ESCC tissues from different clinical stages.

According to the GEPIA database (based on the TCGA database), expression of RNF168 is altered in many tumors, and is increased in ESCC (Figure 1A). Data from the Linkedomics database (based on the TCGA database) (number of ESCC cases = 182, number of normal tissues = 286) demonstrated that RNF168 is increased in ESCC (P<0.05) (Figure 1B). The relationship between pathological staging and RNF168 expression in esophageal squamous cell carcinoma samples was analyzed using the TCGA database, and the results showed that expression of RNF168 in STAGE II was higher than in other stages (Figure 1C).

### RNF168 expression levels in ESCC correlate with tumor size, depth of invasion and pathological stage

**Figure 2:**
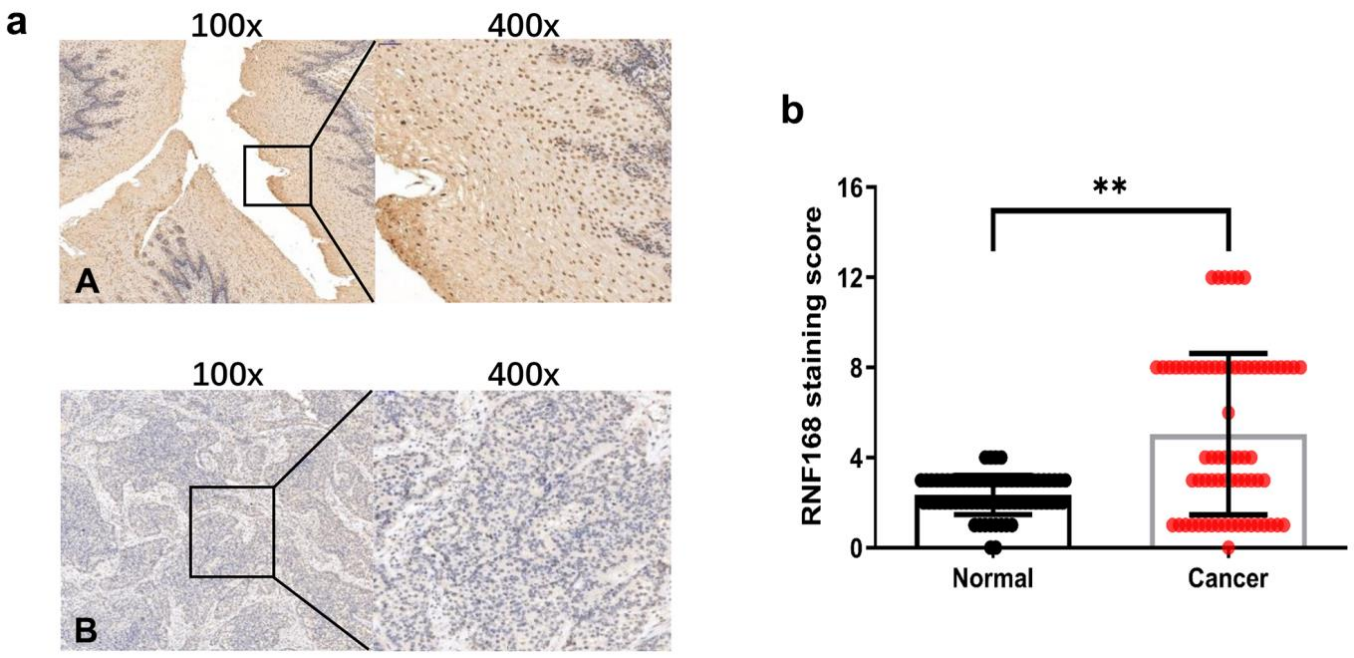
(**a**) Representative results of immunohistochemical staining for RNF168 in human ESCC (**A**) Cancer tissue with high RNF168 expression; (**B**) Cancer tissue with low RNF168 expression); (**b**) RNF168 protein expression scores for esophageal squamous cell carcinoma tissues and matched adjacent normal tissues (** P<0.01).

To study the correlation between RNF168 expression and clinical features of ESCC, we analyzed by immunohistochemistry 69 pairs of ESCC tissues collected from patients (Figure 2A). In patients with ESCC, RNF168 expression was not correlated with gender, age, degree of differentiation, tumor size, lymph node metastasis, or distant metastasis, but did correlate with pathological stage and depth of invasion (Table 1). RNF168 expression was high in 38 (49.2%) of the tumor tissues but only in 4 (5.8%) of the adjacent normal tissues (Figure 2B).

**Table 1:** Correlation between RNF168 expression and clinicopathological features in ESCC.

**Clinical parameters**	**Number of cases**	**HighRNF168 (%)**	**X^2 a^**	**P values**
**Gender**				
**Male**	53	29 (54.7)	0.013	0.717
**Female**	16	9 (56.3)		
**Age**				
**≤40**	13	6 (46.2)	1.504	0.351
**41~65**	32	19 (59.4)		
**>65**	24	13 (54.2)		
**Tumor size** **(cm)**				
**<2.5**	21	7 (33.3)	0.714	0.370
**2.5~9**	44	28 (63.6)		
**>9**	4	3 (75)		
**Differentiation**				
**High**	22	12 (54.5)	1.861	0.264
**Medium**	31	19 (61.3)		
**Low**	16	7 (43.8)		
**Infiltrating depth**				
**T_1_+T_2_**	16	7 (43.8)	3.175	0.041 ^җ^
**T_3_+T_4_**	43	31 (72.1)		
**Lymph node metastases**				
**Yes**	46	27 (58.7)	1.145	0.614
**No**	22	11 (50)		
**Distant metastases**				
**Yes**	6	3 (50)	0.433	0.432
**No**	63	35 (55.6)		
**Pathological stage**				
**I** **II**	6 26	2 (33.3) 14 (53.8)	12.060	0.017 ^ӝ^
**III** **IV**	31 6	21 (67.7) 1 (16.7)		

^a^Pearson’s chi-square test was used for the assay. Significant at P<0.05.

### RNF168 knockdown inhibits tumor cell proliferation, apoptosis, and migration

**Figure 3:**
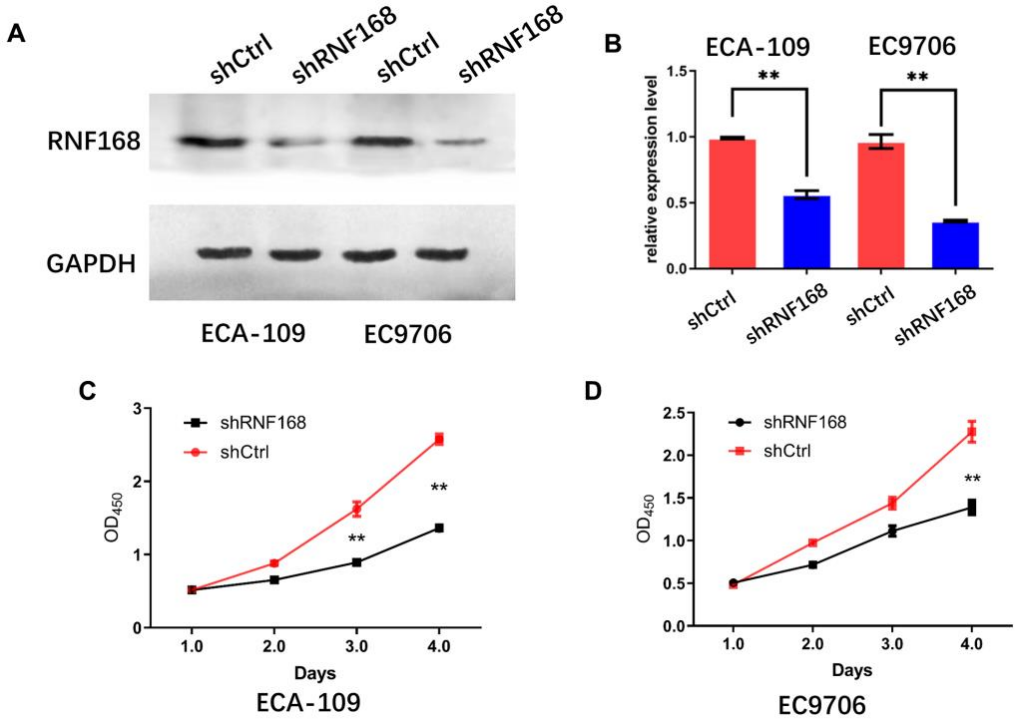
**Effects of RNF168 knockdown on the *in vitro* proliferation of esophageal cancer cells.** (**A**) Western blot and (**B**) qPCR demonstrated that RNF168 was successfully knocked down in ECA-109 and EC9706 cells; (**C**, **D**) cell proliferation measured by the CCK-8 assay. Data are expressed as the mean ± SD of independent experiments. ** P<0.01.

**Figure 4:**
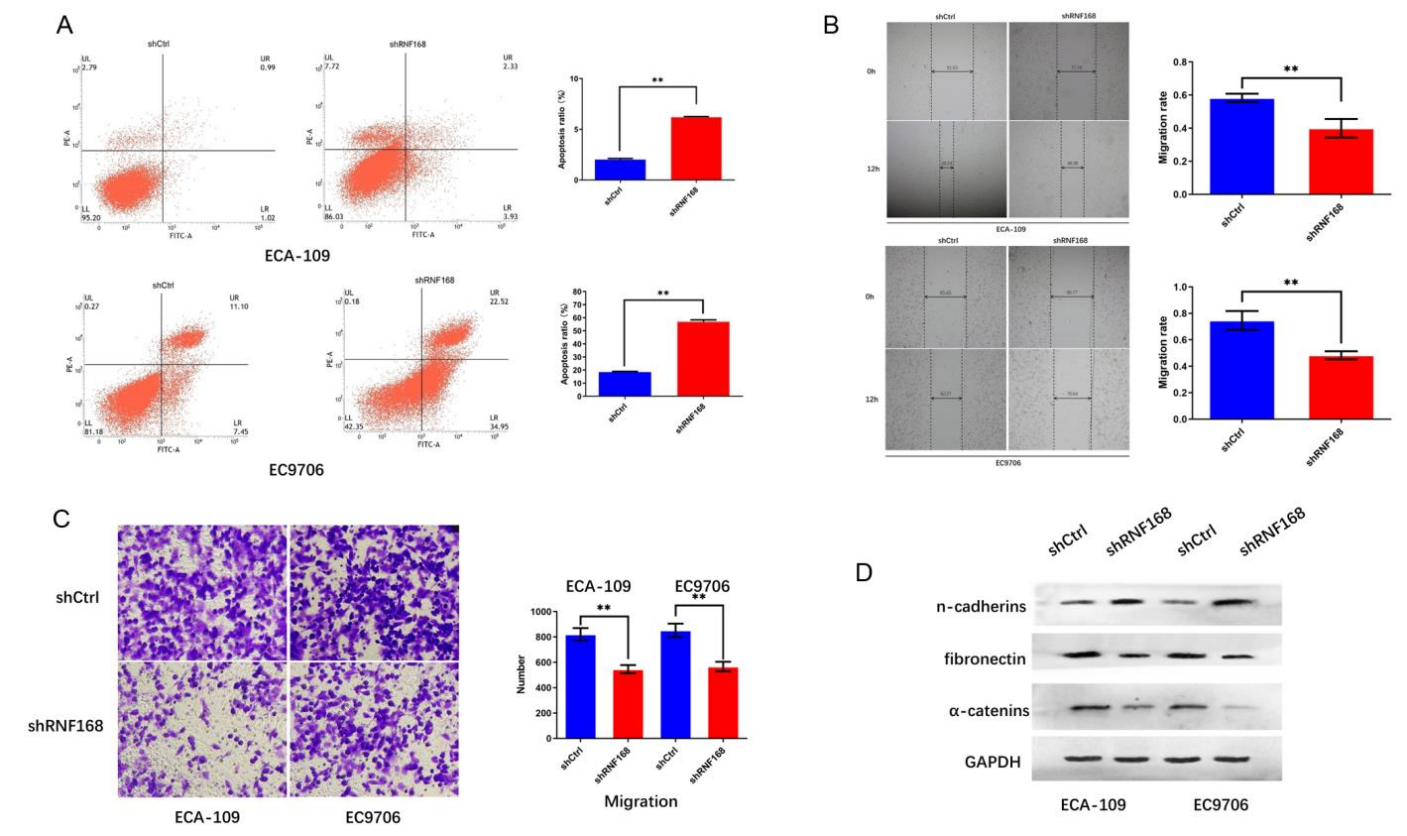
**RNF168 knockdown inhibits apoptosis and migration of esophageal cancer cells.** (**A**) Apoptosis of ECA-109 and EC9706 cells determined by flow cytometry after transduction with shCtrl or ShRNF168 lentiviruses; (**B**) and (**C**) migration capacity of the cells measured by the scratch assay (width in μm) and the transwell assay; (**D**) Expression levels of n-cadherins, fibronectin and α-catenins determined by Western blot. Data are expressed as the mean ± SD of independent experiments. * p<0.05, ** P<0.01.

To further investigate the effects of RNF168 on the biological activity of esophageal cancer cells, we knocked down RNF168 expression in ECA-109 and EC9706 cells by lentiviral transduction (Figures 3A, 3B). We first tested the effects of RNF168 on the proliferation of these two cell lines by means of the CCK-8 assay. We found that proliferation was significantly reduced in the RNF168-knockdown groups (Figures 3C, 3D). The effect of RNF168 on the survival of tumor cells was evaluated by flow cytometry-based apoptosis detection. The numbers of early and late apoptotic cells were analyzed and the results demonstrated that RNF168 knockdown significantly increased apoptosis when compared with the control group (Figure 4A). Scratch and transwell assays demonstrated that RNF168 knockdown impeded migration of ECA-109 and EC9706 cells (Figures 4B, 4C). We also evaluated the correlation between RNF168 expression and epithelial-mesenchymal transition (EMT) in esophageal cancer cells, and found that RNF168 knockdown significantly increased the expression of epithelial n-cadherins, but reduced expression of fibronectin and α-catenins, the mesenchymal markers (Figure 4D).

### RNF168 knockdown suppressed the Wnt/β-catenin signaling pathway

**Figure 5:**
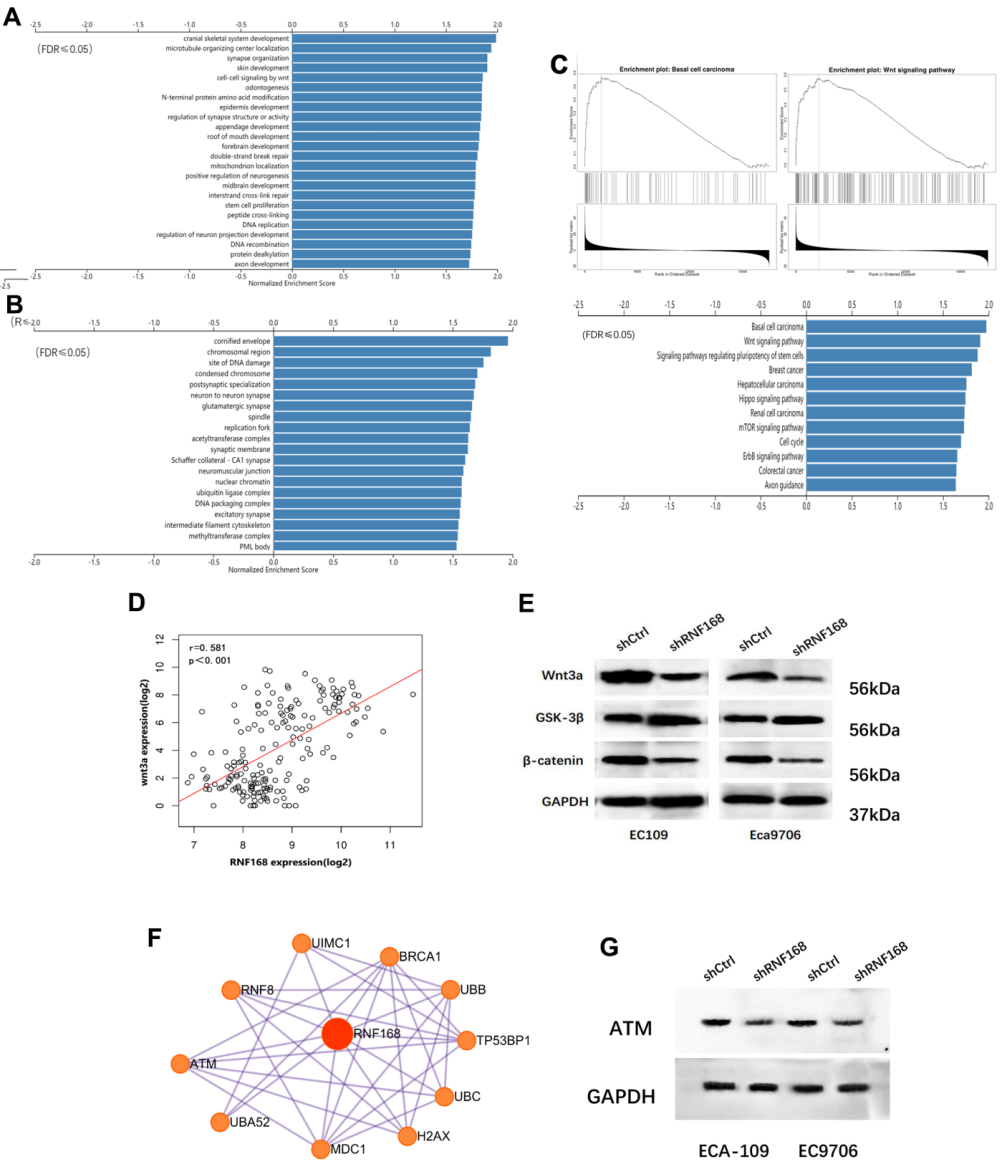
(**A**) and (**B**) Enrichment analysis of differentially expressed genes in 184 cases of esophageal cancer from the TCGA database; (**C**) Pathway enrichment analysis; (**D**) correlation between WNT3A and RNF168 mRNA levels; (**E**) Wnt/β-catenin signaling pathway protein component levels in ECA-109 and EC9706 cells after transduction; (**F**) PPI network for RNF168. (**G**) RNF168 and ATM expression by Western blot.

To further explore how RNF168 contributes to ESCC, we searched the TCGA database, and analyzed 20,140 background genes in 184 cases of esophageal cancer with the LinkedOmics online data analysis tool. Through GSEA (Gene Set Enrichment Analysis) and alignment against the Gene Ontology gene terms, we found that the differentially expressed genes were mainly enriched in the following biological processes: brain development, microtubule organizing center, Wnt signaling pathway, and double-strand break repair (Figure 5A), whereas the cellular components involved were chromosomes and damaged DNA (Figure 5B). We also performed pathway enrichment analysis and found that the differentially expressed genes were enriched in the following pathways: basal cell tumors, wnt signaling, and stem cell signaling (Figure 5C). Pearson correlation analysis suggested that RNF168 was significantly correlated with WNT3A (Figure 5D, r=0.581, p<0.001). To verify these results, we performed Western blot assays to measure the levels of several key proteins in the classic Wnt/β-catenin signaling pathway, and found that upon RNF168 knockdown, WNT3A and β-catenin were inhibited, whereas glycogen synthase kinase 3β (GSK-3β) was activated. Hence, it was concluded that RNF168 may participate in the regulation of classic Wnt/β-catenin signaling in esophageal cancer (Figure 5E). A PPI network (protein protein interaction network) for RNF168 was plotted based on the String-DB database, and this analysis showed that RNF168 mutual effects with RNF8, ATM, H2AX, BRCA1 and TP53BP1, among others (Figure 5F). A Western blot was performed to verify that silencing RNF168 activated the expression of ATM in esophageal squamous cell carcinoma cells (Figure 5G).

### Loss of RNF168 inhibits ESCC carcinogenesis *in vivo*

**Figure 6:**
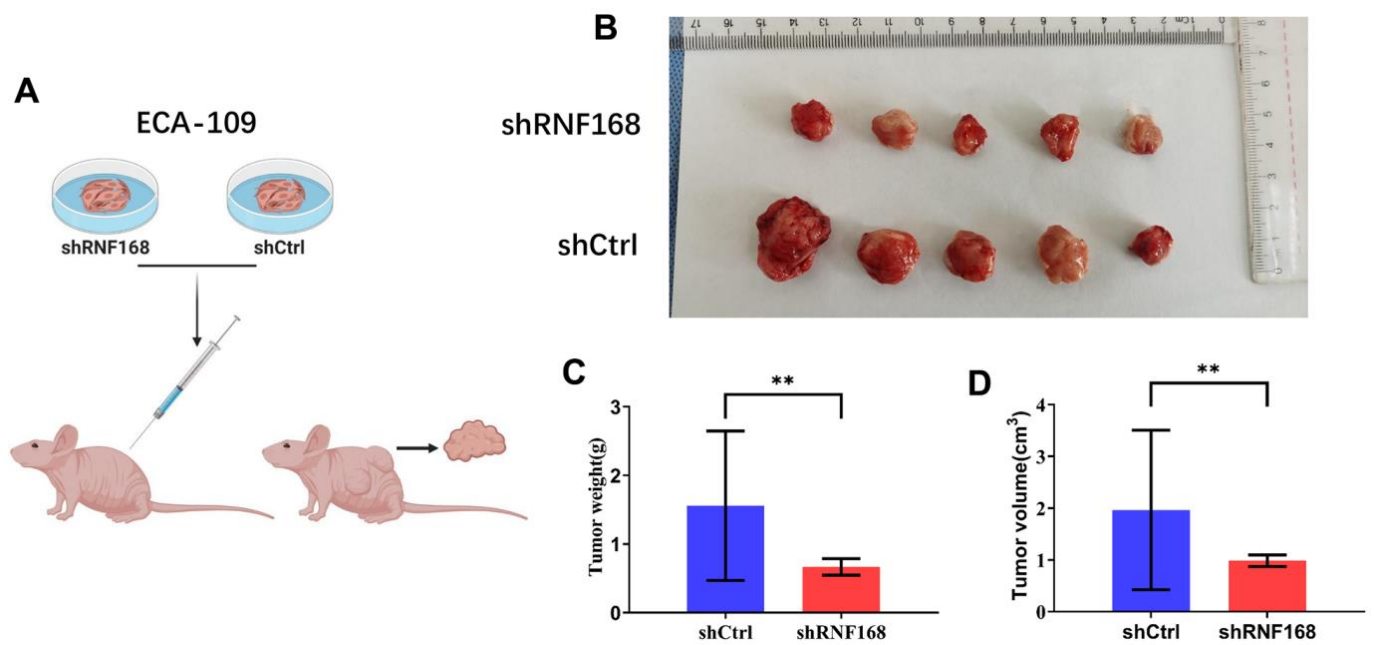
(**A**) RNF168-silenced and control Eca-109 cells were injected into nude mice; (**B**) Gross appearance of the tumors in nude mice injected with ECA-109/shCtrl or ECA-109/RNF168 cells. Weight (**C**) and volume (**D**) of tumors in nude mice transplanted with ECA-109/shCtrl or ECA-109/RNF168 cells. **, P<0.01.

Next, we evaluated the effects of RNF168 depletion on ESCC carcinogenesis *in vivo*. ECA-109 cells transduced with lentiviruses encoding shRNF168 or shCtrl were subcutaneously inoculated into nude mice (Figure 6A). Tumors grew in all mice four weeks after injection (Figure 6B). However, the net weight of the tumors originating from RNF168-silenced cells was significantly less than the weight of the control tumors (Figure 6C, **, P<0.01). As shown in Figure 6D, the growth of tumors originating in RNF168-silenced cells was significantly slower at different time points when compared with the control tumors.

### High expression of RNF168 may influence the survival time of patients with esophageal squamous cell carcinoma

**Figure 7:**
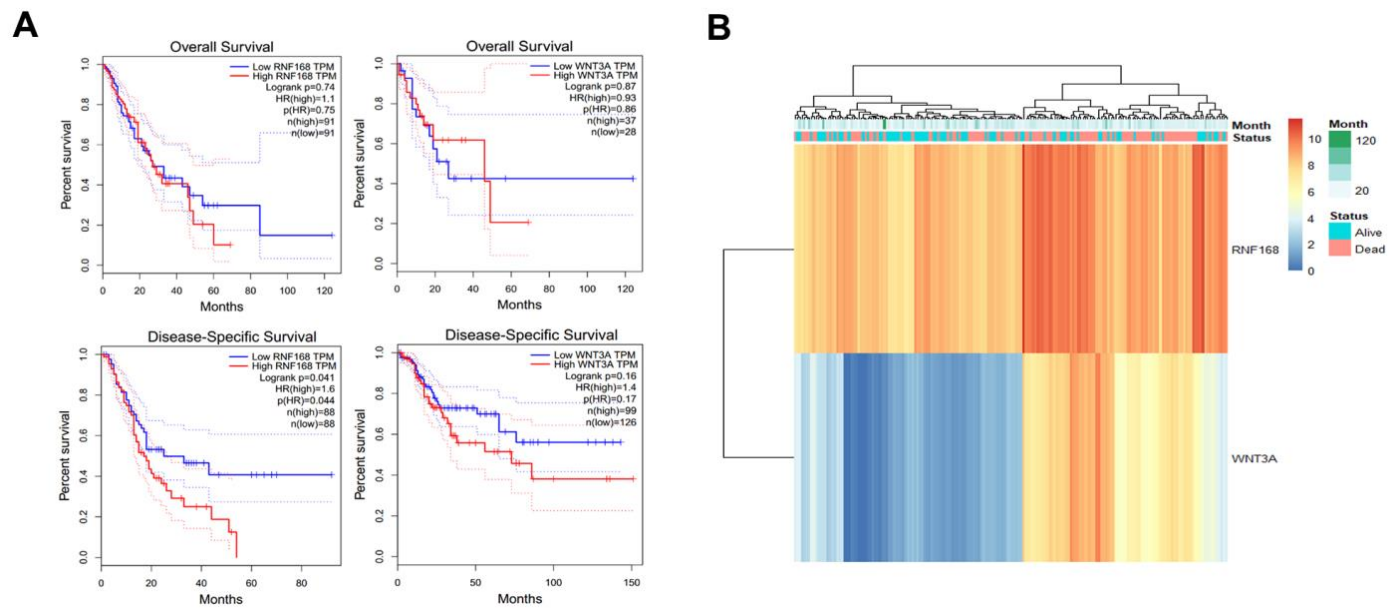
(**A**) TCGA database analysis of RNF168 and WNT3A mRNA expression levels and OS and DSS curves in patients with esophageal squamous cell carcinoma; (**B**) heat maps showing WNT3A and RNF168 expression levels and survival in 184 patients.

The R package pheatmap (v1.0.1) was used to analyze the data of 184 patients with esophageal cancer in the TCGA database. The expression trends for RNF168 and WNT3A and the survival status of the patients were analyzed. It was found that RNF168 and WNT3A mRNA levels were negatively correlated with the survival rate (overall survival and disease-specific survival) of patients with esophageal squamous cell carcinoma (Figure 7A). Heatmaps showed the relationship between RNF168 and WNT3A expression and survival in 184 patients with esophageal cancer (Figure 7B).

## DISCUSSION

Our results showed that RNF168 expression was elevated in ESCC, which was consistent with data from the TCGA database. Notably, RNF168 expression levels correlated with clinicopathological features of the tumor, such as pathological staging and depth of invasion (Table 1), indicating that RNF168 may serve as a biomarker of malignancy in ESCC.

Most patients with esophageal cancer are not diagnosed until the disease is already at an advanced stage, and generally have a poor prognosis despite prompt treatment with surgery, chemoradiotherapy, and targeted drugs [5, 13]. In this study, we found that high RNF168 expression correlated with poor survival of the patients, representing a potential risk factor for patients with ESCC.

In response to DNA damage, a series of ubiquitination processes activate different pathways involved in DNA damage signaling and DNA repair [14]. A double-strand break (DSB) is a special type of DNA damage, and DSB repair requires phosphorylation of MDC1 by ATM and recruitment of RNF8, which functions together with UBC13 to promote ubiquitination of chromatin associated proteins. RNF168 is recruited to the site of DSB by these proteins and participates in the repair of damaged DNA [15–17]. However, overexpression of RNF168 may trigger abnormal repair mechanisms, imbalancing this process and causing cancer [18]. Functional enrichment analysis revealed that RNF168 overexpression may cause changes in Wnt signaling and DSB repair, and the related cellular components were chromosomes and sites of DNA damage, suggesting that RNF168 is mainly localized in the nucleus and plays a major role in DSB repair.

In this study, we also knocked down RNF168 expression to see how this influenced the malignant behavior of esophageal cancer cells, and found that proliferation was significantly inhibited. PPI network analysis revealed that RNF168 may activate with ATM, and participate in the metabolism, growth, and response of cells to oxidative stress. Previous studies have shown that ATM is mainly involved in two pathways, the classic one being the activation of DNA damage checkpoints in response to DSB [19]. Hence, we speculated that RNF168 may participate in this process by ATM, and therefore activate the physiology and proliferation of tumor cells. However, whether ATM is a target of RNF168 requires further verification.

The classic Wnt/β-catenin signaling pathway has been a focus of research in the areas of tumorigenesis and development. Starting with the Wnt ligand, a secreted cysteine-rich glycoprotein, the Wnt signaling pathway plays a major role in embryonic development and tumorigenesis. Classical Wnt signaling is mediated by β-catenin and regulates cell proliferation, differentiation, and migration [20]. In the absence of Wnt ligand, β-catenin is rapidly degraded by the glycogen synthase kinase 3β (GSK3β)-containing destruction complex. It’s widely recognized that GSK3β is inhibited upon Wnt binding, leading to accumulation of β-catenin, which acts as a transcription factor to promote expression of a variety of genes capable of inducing EMT [21]. In our study, RNF168 knockdown inhibited fibronectin and α-catenins expression and activated n-cadherins expression, suggesting induction of EMT. Therefore, we speculated that RNF168 may induce EMT and activate metastasis and other malignant behaviors of ESCC cells by regulating Wnt signaling. Basaloid squamous cell carcinoma (BSCC) of the esophagus is a rare subtype of ESCC [22], which has an especially poor prognosis possibly because it’s more invasive [23]. It has been demonstrated that classic Wnt signaling may also play a role in the occurrence and progression of BSCC [24]. In this study we demonstrated, through pathway enrichment analysis, that RNF168 is correlated with BSCC and Wnt signaling, suggesting that RNF168 may contribute to the malignant behavior of BSCC cells by regulating the Wnt signaling pathway (Figure 5C). Hence, targeting RNF168 and Wnt/β-catenin signaling simultaneously may be a promising strategy to treat this subtype of ESCC.

In summary, our study demonstrates for the first time that RNF168 is highly expressed in human ESCC and that it correlates with the depth of tumor invasion and pathological staging, suggesting its potential use as a marker of ESCC malignancy. Our results also show that RNF168 knockdown interferes with the proliferation and migration of esophageal cancer cells, and suppresses Wnt/β-catenin signaling. Therefore, RNF168 is a promising marker for the diagnosis of ESCC and a potential target for the treatment of this disease.

## MATERIALS AND METHODS

### Clinical data and immunohistochemistry

Paraffin-embedded specimens of pathologically confirmed ESCC and pericancer tissues were collected from 69 patients who underwent radical resection at Gansu Provincial Hospital from January 2016 to December 2018. The 69 patients included 53 males and 16 females, and their average age was 43.71±3.8 (34-76) years. There were no significant differences in terms of gender or age between the groups. None of the patients had received chemoradiotherapy or neoadjuvant therapy before surgery. Informed consent by the patients and approval by the ethics committee were obtained before the study. Expression of RNF168 in cancerous and pericancer tissues was assessed by immunohistochemical staining. Briefly, the histological sections were dewaxed and hydrated, boiled in EDTA antigen retrieval buffer (PH9.0) for 10 min, treated with 3% hydrogen peroxide solution to block internal peroxidase, and incubated with normal goat serum for 30 min to block non-specific binding. Afterwards, anti-RNF168 primary antibody was added (1:50), followed by overnight incubation at 4° C. Next, the slides were allowed to warm to room temperature for 30 min, and incubated with the proper HRP-conjugated secondary antibody (1:200) at room temperature for 50 min. Finally, the antigens were detected by adding DAB and counterstaining with hematoxylin. Brown-yellow particles were considered indicative of RNF168 expression. The quantity of positively stained cells was scored as follows: 0 for <10%, 1 for 10-25%, 2 for 26-50%, 3 for 51-75%, and 4 for >75%. The staining intensity was scored as follows: 0 for no staining, 1 for weak staining, 2 for moderate staining, and 3 for strong staining. The final score was calculated by multiplying the quantitative score by the intensity score (range from 0-12). A final score ≤3 indicated low RNF168 expression, whereas a final score ≥4 was considered high RNF168 expression.

### Cell culture

Esophageal cancer cell lines ECA-109 and EC9706 were purchased from Xiangya Medical College of Central South University and cultured in RPMI 1640 medium (Gibco, Carlsbad, CA, USA) supplemented with 10% fetal bovine serum (Gibco) and 1 % Penicillin/Streptomycin (Beyotime, Shanghai, China). Both cell lines were derived from specimens of patients with ESCC. All cells in this study were used within 6 months after revival of the frozen original culture and were maintained in a 37° C environment containing 5% carbon dioxide.

### Cell transduction

The RNF168-knockdown and control lentiviruses were constructed by Shanghai Genomeditech Co., Ltd. Cells in logarithmic growth phase were detached from the plate by adding 0.25% trypsin, resuspended in complete medium, seeded in a 35mm petri dish at 5 x 10^5^ cells per well, and cultured overnight. Transduction was performed when the cells reached 80% confluence. Briefly, 2μg of the virus stock was diluted in 200μl of serum-free DMEM in a 1.5ml EP tube and the virus solution was incubated at room temperature for 15-20min. The diluted virus was evenly added to the cells in fresh medium, drop by drop, and the cells were placed in a CO_2_ incubator.

### Real-time quantitative PCR (qRT-PCR)

RNF168 shRNAs sequences were shown here: RNF168 shRNA:5’-GGCGAGTTTATGCTGTCCCT-3’, control shRNA sequences were shown: 5’-GCCAGAGGCCACTTGTGTAG-3’.To extract total RNA, the cells were lysed with Trizol reagent at room temperature for 5min in an EP tube. After homogenization, 0.2ml of chloroform was added and capped EP tubes thoroughly mixed by inverting them up and down for 15min, followed by centrifugation. Then, the upper aqueous phase was transferred to a new EP tube, 0.5ml of isopropanol was added, the solution was gently mixed, left at room temperature for 10 minutes, and centrifuged again. cDNA was synthesized from total RNA by using the PrimeScript-RT kit with a gDNA eraser (Takara Biomedical Technology, Dalian). SYBR premix Ex-Taq kit (Takara Biomedical Technology) was used for quantitative real-time PCR analysis.

### Western blot

PVDF membrane was used for protein transfer and probed with antibodies against human RNF168(Rabbit Anti-RNF168 antibody, Cat No:ab229271). GAPDH(Anti-GAPDH antibody, Cat No:ab9485). Fibronectin(Rabbit Anti-Fibronectin antibody, Cat No:ab2413). N-Cadherin(Rabbit Anti-N Cadherin antibody, Cat No:ab76011).α-Catenin(Rabbit Anti-α Catenin antibody, Cat No:ab32572).Wnt3a(Rabbit Anti-Wnt3a antibody, Cat No:ab219412).GSK3β(Rabbit Anti-GSK3 beta antibody, Cat No:ab32391).β-Catenin(Rabbit Anti-beta Catenin antibody, Cat No:ab32572).ATM(abbit Anti-ATM (phospho S1981) antibody, Cat No:ab81292).HRP-conjugated goat anti-rabbit IgG (Abcam, Cat No:ab205718)was used as secondary antibody to detect the proteins. Stably transducted cells were removed from the incubator, washed twice with pre-chilled PBS, lysed on ice with IP lysis buffer, and 5x loading buffer was added. The mixture was heated in boiling water for 10min, cooled on ice, and loaded onto the SDS-PAGE gel wells. Protein concentration was determined by means of a protein quantification kit (BCA method), as instructed by the manufacturer. A total of 200μl of the lysate was mixed with 30μl of 5x loading buffer in a 200μl EP tube, heated in a thermocycler at 98° C for 5min, and cooled on ice for 5min. After 3 such heat-cool cycles, the samples were stored at -80° C as backup. To prepare the SDS-PAGE gel, a 10% separating gel was added, layered with absolute ethanol, and polymerization was allowed to proceed at room temperature for 30min. Freshly prepared 5% stacking gel was added to the top of the separating gel and the comb was inserted. The protein samples were removed from the -80° C freezer, denatured again at 95° C for 3min, placed on ice for 5min to cool them down, and 20μg of each sample was loaded into the gel. Electrophoresis was run at 80V for 30min until the samples reached the resolving gel, then at 120V until the marker bands above and below the target protein band were properly separated. The protein bands were then transferred to a PVDF membrane, which was incubated in 5% skim milk/TBST to block non-specific binding, incubated with primary and secondary antibodies, and then visualized by using the ECL method.

### Cell proliferation assay

Transfected cells in the logarithmic growth phase were detached from the plate with 0.25% trypsin and single cell suspensions counted with CCK-8. Afterwards, cells were seeded into a 96-well plate (10,000 cells per well) and cultured at 37° C under 5% CO2 and 95% humidity. Every 24 hours, 10 μl of CCK-8 was added to each well and, after two hours of incubation, the absorbance at 450 nm was measured using a microplate reader.

### Apoptosis assay

Logarithmic growth phase cells were seeded into 6-well plates. After 24 hours of culture, the cells had adhered completely to the plates. Cells were then trypsinized, collected, rinsed with PBS and fixed overnight with 75% alcohol. About 100uL of cell suspension was retained. One ml of PI reagent was added for staining, followed by flow cytometry analysis. The effects of shRNF168 and shCtrl on apoptosis of ESCC cells were analyzed.

### Transwell and scratch assays

Transwell assays were performed to investigate the migratory ability of esophageal cancer cells. Cell suspensions in serum-free 0.1% BSA-RPMI 1640 (1×10^5^ cells/well) were seeded into migration chambers, which were placed in 24-well plates, and complete culture medium added to each bottom well. After 24 hours of incubation, the chamber membranes were fixed with formalin and stained with crystal violet. Cells on the upper surface of the membrane were removed, and cells beneath the membranes were counted to evaluate their migration ability. For the scratch assay, cells were seeded in 96 well plates at 5x10^4^/well and incubated overnight. The scratches were made with specialized tool. The cells were washed with normal culture medium first, and then with medium supplemented with only 1% FBS. Images were recorded at 0h and 12h with a 100x microscope. Gap distance was measured using Adobe Photoshop CS6 (v13.0).

### Mouse subcutaneous xenograft model

All experimental procedures were approved by the Animal Ethics Committee of Gansu Provincial Hospital. ECA-109 cells transduced with shRNF168 or shCtrl lentiviruses were incubated for 72 hours and then treated with 2 μg/ml puromycin (Clontech) for 96 hours. Puromycin-resistant cells were harvested and injected subcutaneously into the right dorsal flank of 4-week-old female BALB/c nude mice (n=5 per group). Mice were sacrificed after four weeks, and the tumors were isolated for further analysis.The tumor volume (mm^3^) was calculated using the formula = (π/6) ×length× (width)^2^, where the length and width were the longest and shortest diameters, respectively.

### Bioinformatics analysis

Bioinformatics analysis was performed with GEPIA (Gene Expression Profiling Interactive Analysis), an online tool developed by Peking University (http://gepia.cancer-pku.cn) for data analysis based on the cancer genome atlas (TCGA) and the Genotype-Tissue Expression (GTEx) databases. This tool is mainly used for analysis of gene expression in different tumors and to study the correlation between differentially expressed genes and clinical data. In this study, we analyzed expression of RNF168 in ESCC tissue and adjacent normal tissue, as well as the correlation between RNF168 expression and clinical pathology. We also analyzed the correlation between RNF168 mRNA expression and ESCC clinical stage using a third-party tool, the LinkedOmics software (http://www.linkedomics.org/login.php) [25], and evaluated the significance of RNF168 expression for the overall survival of the patients. Functional enrichment and pathway enrichment analyses were performed using the TCGA and GDAC databases, and the correlation between WNT3A expression and RNF168 expression was analyzed based on Pearson’s correlation coeficient. The protein-protein interaction (PPI) network for RNF168 was analyzed using the String-DB database (https://string-db.org).

### Statistical analysis

Data analysis was performed using SPSS 21.0. Two-tailed Student’s t-test was used to compare mean values. Categorical data were analyzed using either the Fisher exact test or the χ2 test. Survival analysis was performed using the Kaplan-Meier method and the log-rank test. Cox proportional hazard regression analysis was used to calculate the relative risk ratios. P < 0.05 was considered statistically significant.
